# Effects of marital status on breast cancer survival by age, race, and hormone receptor status: A population‐based Study

**DOI:** 10.1002/cam4.2352

**Published:** 2019-07-02

**Authors:** Zhen Zhai, Fang Zhang, Yi Zheng, Linghui Zhou, Tian Tian, Shuai Lin, Yujiao Deng, Peng Xu, Qian Hao, Na Li, Pengtao Yang, Hongtao Li, Zhijun Dai

**Affiliations:** ^1^ Department of Breast Surgery, The First Affiliated Hospital College of Medicine Zhejiang University Hangzhou China; ^2^ Department of Oncology The Second Affiliated Hospital of Xi'an Jiaotong University Xi'an China; ^3^ The Second Department of Oncology The Central Hospital of Enshi Tujia and Miao Autonomous Prefacture Enshi China; ^4^ Department of Breast Head and Neck surgery The 3rd Affiliated Teaching Hospital of Xinjiang Medical University (Affiliated Tumor Hospital) Urumqi China

**Keywords:** breast cancer, marital status, prognosis, SEER

## Abstract

**Introduction:**

It remains unclear whether marital status could affect the breast cancer‐caused special survival (BCSS) of patients with breast cancer. Therefore, we sought to explore the influence of demographic and pathological factors on prognosis of patients with breast cancer.

**Materials and methods:**

We selected patients meeting the eligibility criteria from the Surveillance, Epidemiology, and End Results (SEER) cancer registry program. We assessed the effect of marital status on overall survival (OS) and BCSS using Kaplan‐Meier curve and multivariate Cox proportional hazards regression.

**Results:**

Compared with divorced/separated/widowed (DSW) patients, the married (AHR 0.7483, 95% CI: 0.729‐0.7682, *P* < 0.001) and single patients had better BCSS (AHR 0.9096, 95% CI: 0.8796‐0.9406, *P* < 0.001). Married patients kept better prognosis among all age subgroups, while the better BCSS of single patients occurred only in groups older than 35 years. As for race and hormone receptor status (HRs), the better BCSS of single patients was only observed in white race (AHR 0.881, 95% CI: 0.8457‐0.9177, *P* < 0.001) and patients with ER+/PR + status (AHR 0.8844, 95% CI: 0.8393‐0.932, *P* < 0.001).

**Conclusion:**

Our findings demonstrated that married and single patients with breast cancer had better prognosis than their DSW counterparts. Age, race, and HRs could affect the correlation between marital status and BCSS.

## 1 INTRODUCTION

Psychosocial factors are associated with the outcome of cancer patients. It can be said with certainty that cancer patients could obtain better prognosis from some psychosocial factors like coping strategies, emotional support, and social integration.^1^, ^2^ Marriage is one of the most important forms of social relations influencing on cancer patients. Previous studies have demonstrated that marital status could affect the survival outcome of several kinds of cancers, and it might act as an independent prognostic factor for overall survival (OS) in patients with breast cancer.^3^, ^4^, ^5^ Married patients were considered to have more emotional and financial support, which helps them to be diagnosed at earlier stage and receive proper treatments with better adherence, then finally prolong their overall survival.^6^, ^7^, ^8^, ^9^ However, further analysis of marital subgroups was neglected, which might reveal the potential mechanism generating the influence of marital status on prognosis.

Breast cancer is the most frequently diagnosed cancer for women worldwide and is also the leading cause of cancer death for female patients in over 100 countries.^10^ The incidence of breast cancer in the US population is expected to reach 30%‐40% by 2020.^11^ As a systemic disease, the formation of breast cancer is due to complex interaction of psychosocial and physiological factors.^12^ Recently, the association between marital status and OS has been studied for some cancers. These studies proved that marital status acts as an independent prognostic factor for survival in patients with several cancers including breast cancer, and married patients gain a significant survival benefit compared with unmarried patients.^13^, ^14^, ^15^, ^16^, ^17^, ^18^, ^19^, ^20^ These conclusions suggest us a general relationship between marital status and the survival of breast cancer patients. However, some specific problems still need to be solved. First, most of these studies only took OS into consideration and neglected to investigate cancer‐specific survival, including breast cancer‐caused special survival (BCSS). Second, majority of their data were based on small samples, restricted by population diversity or limited follow‐up time. Moreover, previous studies neglected to explore the difference among the unmarried groups and whether the effect of marital status on prognosis differs across patients' demographic and pathological subgroups.^15^, ^17^ Among these, age and hormone receptor status (HRs) were reported as two key independent prognostic factors for breast cancer survival.^21^

In view of present research station, we conducted this analysis to explore the correlation between marital status and BCSS and whether the association varied by age, race, and HRs. Our study was based on the data provided by Surveillance, Epidemiology, and End Results (SEER) cancer registry program. The SEER database includes population‐based data from 18 cancer registries in population‐based catchment areas related to cancer diagnoses, treatment and survival in approximately 30% of the population in the United States from 1973 to 2014.^22^

## 2 MATERIALS AND METHODS

### 2.1 Patient selection and data extraction

To estimate the correlations between marital status and BCSS in patients, we used SEER*Stat 8.3.4 software to extract eligible patients included in the database. We enrolled a total of 476 028 patients diagnosed with breast cancer, who were aged 18 years or above at diagnosis, between 2004 and 2012. After excluding cases diagnosed using autopsy or death certificates only, we selected 298 434 patients according to the following criteria: (a) only one primary malignancy in their lifetime; (b) limited to the following histological types, according to the International Classification of Diseases for Oncology, 3rd Edition (ICD‐O‐3): 8500, 8501, 8510, 8512, 8513, 8514, 8520, 8521, 8522, 8523, 8524, 8525, 8530, and 8541; (c) known race; (d) known marital status; (e) known grade; (f) known stage (Breast Cancer Adjusted AJCC Cancer Staging Manual 6th Edition); (g) known surgery situation; and (h) known survival months after diagnosis. We excluded patients for whom the aforementioned data were missing. Marital status at diagnosis was the primary variable of interest, and participants were classified as married, single, and divorced/separated/widowed (DSW), which were considered as unfavorable marital status. The access to and use of SEER data did not require informed patient consent, and all procedures were performed in accordance with approved guidelines. The Ethics Committee of the Second Affiliated Hospital of Xi'an Jiaotong University approved this study.

### 2.2 Statistical analysis

Descriptive statistics were performed to investigate baseline characteristics of the patient population. Patients' clinical characteristics were compared between different categories of marital status using the Chi‐squared test. We evaluated the OS and BCSS rates among different categories of marital status using Kaplan‐Meier curves and the log‐rank tests. For multivariate analysis, multivariate Cox proportional analyses were used to calculate hazard ratios (HR) and their corresponding 95% confidence intervals (CI), to assess the influence of marital status on overall and cancer‐specific survival. We also explored the effect of marital status according to age, race, and HRs using Cox regression analyses and calculated the adjusted hazard ratio (AHR) to assess the survival difference among marital subgroups in special demographic or pathological subgroups. A two‐sided *P* value of less than 0.05 was determined to be statistically significant. All statistical analyses were performed and figures were created using R 3.5.3 software (R Foundation for Statistical Computing, Vienna, Austria).

## 3 RESULTS

### 3.1 Patients' baseline characteristics

**Figure 1:**
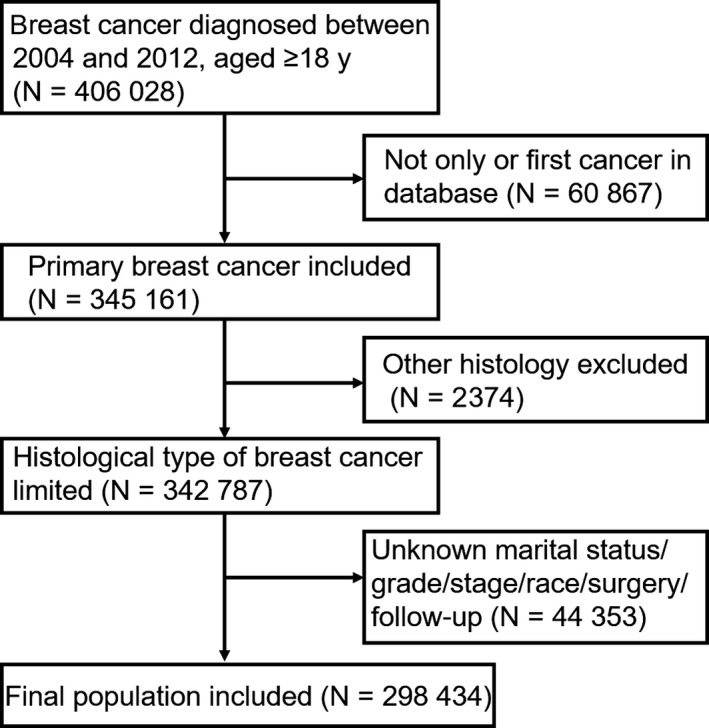
Flowchart for included patients from the SEER

In view of the inclusion criteria, this study included 298 434 patients diagnosed with breast cancer between 2004 and 2012. A flowchart of the study participant selection process was shown in Figure 1. The 10‐year survival rate for the married, single, and DSW participants was 79.9%, 71.1% and 59%, respectively. As shown in Table 1, there were many more female participants (296 500; 99.4%) than the males (1934; 0.6%), so it was inappropriate to compare clinicopathological characteristics by sex. Among eligible patients, 176 109 (59.0%) were married at diagnosis, 43 262 (14.5%) were single, and 79 063 (26.5%) were DSW. The rate of being married was higher among patients aged 35‐70 years, as well as among Asian or Pacific Islanders (API), and this rate decreased with higher tumor stage. Black patients, as well as patients younger than 35 years, showed the highest rate of being single. And not surprisingly, the rate of being DSW was highest among patients aged 70 years or older. Compared with the single or DSW, tumors in the married were more inclined to be at stage 0 or I. There was a less proportion of being dead attributable to breast cancer in the married subgroup as well. In addition, married patients received more surgeries either in the females or males, and the females received more breast‐conserving surgery (BCS) (Table 2).

**Table 1:** Baseline clinicopathological characteristics of patients with breast cancer in SEER database

Characteristics	Total	Married	Single	DSW	*P*‐value
Overall	298 434	176 109 (59%)	43 262 (14.5%)	79 063 (26.5%)	
Age (years)					<0.001
<35	6813	3807 (55.9%)	2551 (37.4%)	455 (6.7%)	
35‐70	219 252	142 260 (64.9%)	34 869 (15.9%)	42 123 (19.2%)	
≥70	72 369	30 042 (41.5%)	5842 (8.1%)	36 485 (50.4%)	
Sex					<0.001
Female	296 500	174 763 (58.9%)	42 984 (14.5%)	78 753 (26.6%)	
Male	1934	1346 (69.6%)	278 (14.4%)	310 (16%)	
Race					<0.001
White	241 019	146 707 (60.9%)	30 110 (12.5%)	64 202 (26.6%)	
Black	31 729	11 973 (37.7%)	9720 (30.6%)	10 036 (31.6%)	
AN/AI	1542	794 (51.5%)	307 (19.9%)	441 (28.6%)	
API	24 144	16 635 (68.9%)	3125 (12.9%)	4384 (18.2%)	
Stage					<0.001
Stage 0/I	138 499	85 193 (61.5%)	16 938 (12.2%)	36 368 (26.3%)	
Stage II	108 087	63 388 (58.6%)	16 666 (15.4%)	28 033 (25.9%)	
Stage III	39 362	21 795 (55.4%)	6955 (17.7%)	10 612 (27%)	
Stage IV	12 486	5733 (45.9%)	2703 (21.6%)	4050 (32.4%)	
Grade					<0.001
Grade I	59 689	36 007 (60.3%)	7 424 (12.4%)	16 258 (27.2%)	
Grade II	128 227	75 435 (58.8%)	17 766 (13.9%)	35 026 (27.3%)	
Grade III	107 872	63 108 (58.5%)	17 604 (16.3%)	27 160 (25.2%)	
Grade IV	2646	1559 (58.9%)	468 (17.7%)	619 (23.4%)	
Surgery					<0.001
BCS	163 932	98 869 (60.3%)	22 091 (13.5%)	42 972 (26.2%)	
Other	121 334	71 667 (59.1%)	18 162 (15%)	31 505 (26%)	
No surgery	13 168	5573 (42.3%)	3 009 (22.9%)	4 586 (34.8%)	
HRs					<0.001
ER+/PR+	192 975	115 270 (59.7%)	27 030 (14%)	50 675 (26.3%)	
ER+/PR‐	34 393	19 488 (56.7%)	5008 (14.6%)	9897 (28.8%)	
ER‐/PR+	3223	1951 (60.5%)	531 (16.5%)	741 (23%)	
ER‐/PR‐	54 669	32 027 (58.6%)	8727 (16%)	13 915 (25.5%)	
Other	13 174	7373 (56%)	1966 (14.9%)	3835 (29.1%)	
Cause of death					<0.001
Alive or dead of other cause	266 598	160 869 (60.3%)	37 418 (14%)	68 311 (25.6%)	
Dead (attributable to this cancer)	31 836	15 240 (47.9%)	5844 (18.4%)	10 752 (33.8%)	

Abbreviations: AN/AI, American Indian/Alaska Native; API, Asian or Pacific Islander; BCS, breast‐conserving surgery; DSW, divorced/separated/widowed; ER, estrogen receptor; HRs, hormone receptor status; PR, progesterone receptor.

**Table 2:** Baseline demographic characteristics of patients stratified by marital status (%)

Characteristics	Female, N = 296 500			Male, N = 1934		
	Married	Single	DSW	Married	Single	DSW
	N = 174 763	N = 42 984	N = 78 753	N = 1346	N = 278	N = 310
Age (years)						
<35	2.2	5.9	0.6	0.6	1.4	0.3
35‐70	80.9	80.6	53.3	62.6	75.9	46.8
≥70	16.9	13.4	46.1	36.8	22.7	52.9
Race						
White	83.3	69.6	81.2	83	69.8	82.6
Black	6.8	22.4	12.7	10.3	26.6	15.2
AN/AI	0.5	0.7	0.6	0.4	0	0
API	9.5	7.2	5.6	6.4	3.6	2.3
Stage						
Stage 0/I	48.5	39.3	46.1	33.6	21.9	21.6
Stage II	35.9	38.5	35.4	42.9	44.6	45.2
Stage III	12.3	16	13.4	17.5	20.5	23.2
Stage IV	3.2	6.2	5.1	6	12.9	10
Grade						
Grade I	20.5	17.2	20.6	11.4	13.3	9.7
Grade II	42.8	41	44.3	51.6	51.4	50.3
Grade III	35.8	40.7	34.3	36.4	34.9	38.7
Grade IV	0.9	1.1	0.8	0.5	0.4	1.3
Surgery						
BCS	56.5	51.3	54.5	9.8	11.9	8.7
Other	40.3	41.8	39.7	86.5	77.3	85.2
No surgery	3.2	6.9	5.8	3.7	10.8	6.1
HRs						
ER+/PR+	65.3	62.4	64	81.8	79.9	81.6
ER+/PR‐	11.1	11.6	12.5	9.4	10.4	8.4
ER‐/PR+	1.1	1.2	0.9	0.4	0.7	1
ER‐/PR‐	18.3	20.3	17.7	2.2	3.6	1.9
Other	4.2	4.5	4.8	6.2	5.4	7.1
Cause of death						
Alive or dead of other cause	91.4	86.5	86.4	86.3	77.7	80.3
Dead (attributable to this cancer)	8.6	13.5	13.6	13.7	22.3	19.7

Abbreviations: DSW, divorced/separated/widowed; AN/AI, American Indian/Alaska Native; API, Asian or Pacific Islander; BCS, breast‐conserving surgery; HRs, hormone receptor status; ER, estrogen receptor; PR, progesterone receptor.

### 3.2 Effects of marital status on overall survival and cancer‐specific survival

**Figure 2:**
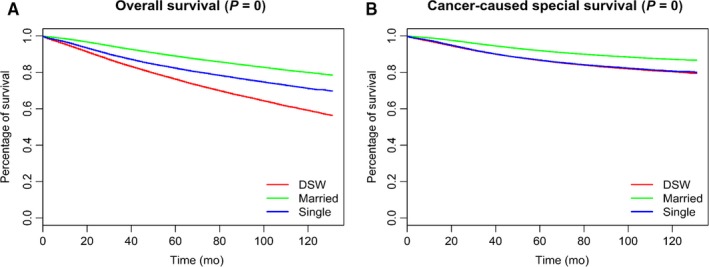
Kaplan‐Meier Survival curves: The overall (A) and cancer‐caused special survival (B) of patients with breast cancer according to marital status

Kaplan‐Meier curves were generated by marital status to estimate overall and cancer‐specific survival of patients with breast cancer. In further log‐rank tests, the married subgroup showed significantly (*P* < 0.001) better OS and BCSS than the unmarried groups, while there was no significant difference of BCSS between the single and the DSW (Figure 2). Considering the possible interaction among variables, we then conducted multivariate Cox regression analysis. It turned out that compared with the DSW, being married and being single were both related to better OS (married: HR 0.6444, 95% CI: 0.6318‐0.6573, *P* < 0.001; single: HR 0.8712, 95% CI: 0.8484‐0.8947, *P* < 0.001) and BCSS (married: HR 0.7483, 95% CI: 0.729‐0.7682, *P* < 0.001; single: HR 0.9096, 95% CI: 0.8796‐0.9406, *P* < 0.001). As for age subgroups, patients aged 70 years or older suffered worse OS (HR 3.3121, 95% CI: 3.2506‐3.3748, *P* < 0.001) and BCSS (HR 1.8478, 95% CI: 1.8005‐1.8963, *P* < 0.001) than patients aged 35‐70 years or younger than 35 years, between which there was no remarkable difference of survival observed. Compared with female patients, male experienced worse OS (HR 1.5298, 95% CI: 1.4112‐1.6584, *P* < 0.001) and BCSS (HR 1.2934, 95% CI: 1.1553‐1.448, *P* < 0.001). In different race subgroups, API patients showed better prognosis, while the black showed worse BCSS (using the white as reference; API: BCSS‐HR 0.7619, 95% CI: 0.7262‐0.7993, *P* < 0.001; black: BCSS‐HR 1.2984, 95% CI: 1.2602‐1.3379, *P* < 0.001). Compared with patients without surgery, better BCSS was observed across those who received BCS (HR 0.3349, 95% CI: 0.322‐0.3483, *P* < 0.001) or other operations (HR 0.4234, 95% CI: 0.4087‐0.4386, *P* < 0.001). In addition, the higher the grade, the worse the prognosis. And similar trends could be found across the stage. Compared with other HRs subgroups, the subgroup of estrogen receptor positive and progesterone receptor positive (ER+/PR+) was associated with better OS and BCSS (Table 3).

**Table 3:** Multivariate analyses of overall and cancer‐caused special survival of patients with breast cancer

Variable	Multivariate				Multivariate		
	OS HR	95% CI	*P*‐Value		BCSS HR	95% CI	*P‐*Value
Age (years)				Age (years)			
35‐70	Baseline			35‐70	Baseline		
<35	0.9767	0.9213‐1.0354	0.4290	<35	0.9914	0.9326‐1.0538	0.7814
≥70	3.3121	3.2506‐3.3748	<0.001	≥70	1.8478	1.8005‐1.8963	<0.001
Sex				Sex			
Female	Baseline			Female	Baseline		
Male	1.5298	1.4112‐1.6584	<0.001	Male	1.2934	1.1553‐1.448	<0.001
Race				Race			
White	Baseline			White	Baseline		
Black	1.2781	1.247‐1.3099	<0.001	Black	1.2984	1.2602‐1.3379	<0.001
AN/AI	1.2703	1.1369‐1.4193	<0.001	AN/AI	1.1988	1.0434‐1.3775	0.0105
API	0.7354	0.7073‐0.7647	<0.001	API	0.7619	0.7262‐0.7993	<0.001
Marital				Marital			
DSW	Baseline			DSW	Baseline		
Married	0.6444	0.6318‐0.6573	<0.001	Married	0.7483	0.729‐0.7682	<0.001
Single	0.8712	0.8484‐0.8947	<0.001	Single	0.9096	0.8796‐0.9406	<0.001
Stage				Stage			
Stage 0/I	Baseline			Stage 0/I	Baseline		
Stage II	1.6699	1.6305‐1.7103	<0.001	Stage II	2.9902	2.8713‐3.114	<0.001
Stage III	3.7727	3.673‐3.8751	<0.001	Stage III	9.0426	8.673‐9.428	<0.001
Stage IV	10.8121	10.4493‐11.1874	<0.001	Stage IV	30.9787	29.5487‐32.478	<0.001
Grade				Grade			
Grade I	Baseline			Grade I	Baseline		
Grade II	1.1837	1.1494‐1.2189	<0.001	Grade II	1.6902	1.6046‐1.7803	<0.001
Grade III	1.5688	1.5215‐1.6177	<0.001	Grade III	2.5914	2.4598‐2.7299	<0.001
Grade IV	1.5395	1.4257‐1.6624	<0.001	Grade IV	2.5379	2.3036‐2.7959	<0.001
Surgery				Surgery			
No surgery	Baseline			No surgery	Baseline		
BCS	0.3433	0.3321‐0.3548	<0.001	BCS	0.3349	0.322‐0.3483	<0.001
Other	0.425	0.4121‐0.4384	<0.001	Other	0.4234	0.4087‐0.4386	<0.001
HRs				HRs			
ER+/PR+	Baseline			ER+/PR+	Baseline		
ER+/PR‐	1.3344	1.3003‐1.3693	<0.001	ER+/PR‐	1.5644	1.5132‐1.6175	<0.001
ER‐/PR+	1.5926	1.4786‐1.7155	<0.001	ER‐/PR+	1.9623	1.8015‐2.1375	<0.001
ER‐/PR‐	1.7221	1.6837‐1.7614	<0.001	ER‐/PR‐	2.1679	2.1089‐2.2285	<0.001
Other	1.4388	1.3878‐1.4917	<0.001	Other	1.6497	1.5719‐1.7313	<0.001

Abbreviations: AN/AI, American Indian/Alaska Native; API, Asian or Pacific Islander; BCS, breast‐conserving surgery; BCSS, breast cancer‐caused special survival; CI, confidence interval; DSW, divorced/separated/widowed; ER, estrogen receptor; HR, hazard ratio; HRs, hormone receptor status; OS, overall survival; PR, progesterone receptor.

### 3.3 Effects of marital status stratified by demographic and pathological subgroups

To further investigate the prognostic effect of marital status on prognosis by different demographic and pathological subgroups, we stratified all cases according to age, sex, race, and HRs and performed multivariate analyses. Compared with the DSW and the single, the better OS of married patients was consistent among most subgroups, although this effect vanished in American Indian/Alaska Native (AN/AI) patients. Compared with the DSW, the significant OS benefit of the single occurred only in patients aged 35 years or older (35‐70 years: AHR 0.8848, 95% CI: 0.8542‐0.9164, *P* < 0.001; ≥70 years: AHR 0.8469, 95% CI: 0.8087‐0.8868, *P* < 0.001) according to age. And the better survival of single patients over the DSW was consistent in the race of white and black, as well as the HRs of ER+/PR+, ER+/PR‐, or ER‐/PR+ (Table 4). As for BCSS, there was no significant difference observed in AN/AI patients among three marital subgroups, while married patients showed better BCSS than DSW patients in most subgroups except for the male. Compared with the DSW, better BCSS of the single was observed only in patients aged 35 years or older (35‐70 years: AHR 0.9313, 95% CI: 0.8944‐0.9697, *P* = 0.001; ≥70 years: AHR 0.8752, 95% CI: 0.8148‐0.94, *P* < 0.001), in race of white (AHR 0.881, 95% CI: 0.8457‐0.9177, *P* < 0.001), or in HRs of ER+/PR+ (AHR 0.8844, 95% CI: 0.8393‐0.932, *P* < 0.001) or ER‐/PR‐ (AHR 0.9202, 95% CI: 0.8693‐0.9741, *P* = 0.004) (Table 5).

**Table 4:** Adjusted hazard ratio for overall survival associated with marital status in different clinicopathological subgroups

OS	Married vs DSW			Single vs DSW			Married vs Single		
Variable	AHR	95% CI	*P*‐value	AHR	95% CI	*P*‐value	AHR	95% CI	*P*‐value
All	0.6444	0.6318‐0.6573	<0.001	0.8712	0.8484‐0.8947	<0.001	0.7396	0.7208‐0.759	<0.001
<35	0.683	0.5484‐0.8507	<0.001	0.8625	0.6911‐1.0765	0.191	0.7919	0.6994‐0.8966	<0.001
35‐70	0.6872	0.6677‐0.7074	<0.001	0.8848	0.8542‐0.9164	<0.001	0.7767	0.7526‐0.8016	<0.001
≥70	0.6076	0.5908‐0.6249	<0.001	0.8469	0.8087‐0.8868	<0.001	0.7175	0.6831‐0.7535	<0.001
Sex									
Female	0.6451	0.6324‐0.6581	<0.001	0.8686	0.8456‐0.8921	<0.001	0.7427	0.7237‐0.7622	<0.001
Male	0.6308	0.51735‐0.7691	<0.001	1.1371	0.87716‐1.4742	0.332	0.5547	0.44385‐0.6933	<0.001
Race									
White	0.6289	0.6154‐0.6428	<0.001	0.8533	0.8266‐0.8809	<0.001	0.737	0.7148‐0.76	<0.001
Black	0.7601	0.7189‐0.8036	<0.001	0.9129	0.8633‐0.9652	0.001	0.8326	0.7871‐0.8808	<0.001
AN/AI	0.872	0.6688‐1.1369	0.311	0.9635	0.693‐1.3395	0.825	0.905	0.6653‐1.231	0.525
API	0.6216	0.5676‐0.6808	<0.001	0.9107	0.8045‐1.0308	0.139	0.6826	0.6112‐0.7624	<0.001
HRs									
ER+/PR+	0.611	0.5943‐0.6281	<0.001	0.8618	0.8294‐0.8954	<0.001	0.709	0.6828‐0.7361	<0.001
ER+/PR‐	0.6389	0.6063‐0.6734	<0.001	0.8673	0.8092‐0.9297	<0.001	0.7367	0.6879‐0.7889	<0.001
ER‐/PR+	0.6483	0.5449‐0.7713	<0.001	0.8364	0.6663‐1.0499	0.123	0.7751	0.629‐0.9552	0.017
ER‐/PR‐	0.7176	0.6908‐0.7455	<0.001	0.8954	0.8516‐0.9414	<0.001	0.8015	0.7648‐0.84	<0.001
Other	0.6301	0.5817‐0.6825	<0.001	0.924	0.836‐1.0213	0.122	0.6819	0.617‐0.7536	<0.001

Abbreviations: AHR: adjusted hazard ratio; AN/AI: American Indian/Alaska Native; API: Asian or Pacific Islander; CI: confidence interval; DSW: divorced/separated/widowed; ER: estrogen receptor; HRs: hormone receptor status; OS: overall survival; PR: progesterone receptor.

**Table 5:** Adjusted HR for cancer‐caused special survival associated with marital status in different clinicopathological subgroups

BCSS	Married vs DSW			Single vs DSW			Married vs Single		
Variable	AHR	95% CI	*P*‐value	AHR	95% CI	*P*‐value	AHR	95% CI	*P*‐value
All	0.7483	0.729‐0.7682	<0.001	0.9096	0.8796‐0.9406	<0.001	0.8227	0.7976‐0.8487	<0.001
Age (years)									
<35	0.6856	0.545‐0.8624	0.001	0.8676	0.6883‐1.0937	0.229	0.7902	0.69431‐0.8993	<0.001
35‐70	0.7774	0.752‐0.8037	<0.001	0.9313	0.8944‐0.9697	0.001	0.8348	0.8057‐0.8649	<0.001
≥70	0.711	0.68‐0.7434	<0.001	0.8752	0.8148‐0.94	<0.001	0.8124	0.753‐0.8764	<0.001
Sex									
Female	0.7488	0.7294‐0.7688	<0.001	0.9071	0.8771‐0.9382	<0.001	0.8255	0.8001‐0.8517	<0.001
Male	7.53E‐01	0.56039‐1.0109	0.059	1.2325	0.8539‐1.779	0.264	0.6277	0.4639‐0.8494	0.001
Race									
White	0.7321	0.7109‐0.7539	<0.001	0.881	0.8457‐0.9177	<0.001	0.831	0.8003‐0.8629	<0.001
Black	0.8455	0.7907‐0.9042	<0.001	0.9888	0.925‐1.057	0.741	0.8551	0.8019‐0.9119	<0.001
AN/AI	1.0282	0.72678‐1.4545	0.875	1.123	0.743‐1.6974	0.582	0.9196	0.6369‐1.3278	0.655
API	0.7073	0.6297‐0.7945	<0.001	0.9508	0.8156‐1.1084	0.519	0.7417	0.6509‐0.8451	<0.001
HRs									
ER+/PR+	0.7175	0.6892‐0.7469	<0.001	0.8844	0.8393‐0.932	<0.001	0.8113	0.7726‐0.8518	<0.001
ER+/PR‐	0.7479	0.6992‐0.7999	<0.001	0.9489	0.8719‐1.0326	0.224	0.7882	0.7277‐0.8537	<0.001
ER‐/PR+	0.6542	0.5355‐0.7991	<0.001	0.8251	0.6382‐1.0667	0.142	0.7928	0.6284‐1.0003	0.05
ER‐/PR‐	0.7916	0.7574‐0.8273	<0.001	0.9202	0.8693‐0.9741	0.004	0.8602	0.8167‐0.906	<0.001
Other	0.731	0.6556‐0.8149	<0.001	0.9839	0.8648‐1.1193	0.805	0.7429	0.6572‐0.8399	<0.001

Abbreviations: AHR, adjusted hazard ratio; AN/AI, American Indian/Alaska Native; API, Asian or Pacific Islander; BCSS, breast cancer‐caused special survival; CI, confidence interval; DSW, divorced/separated/widowed; ER, estrogen receptor; HRs, hormone receptor status; PR, progesterone receptor.

**Figure 3:**
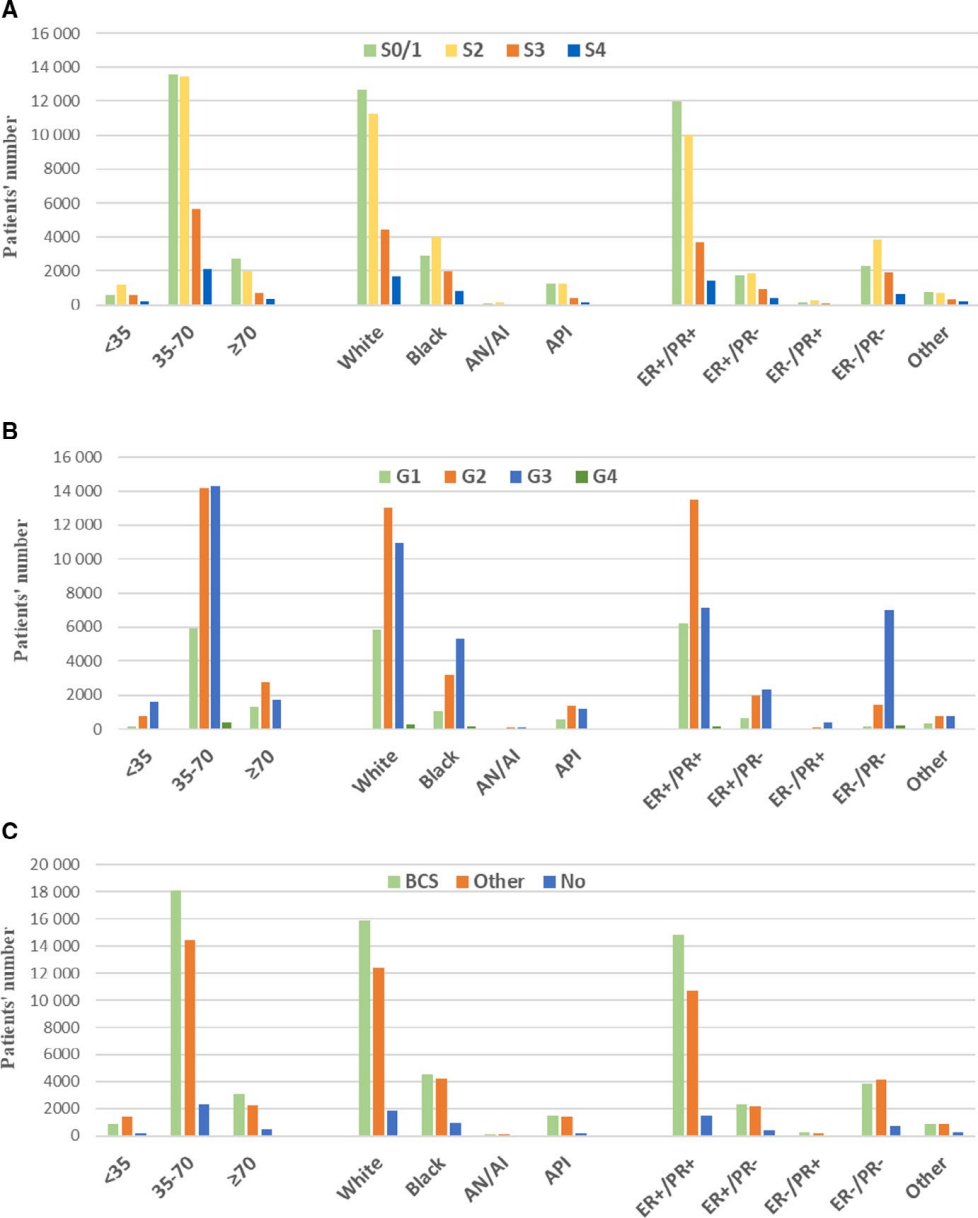
Number of Single patients in different clinicopathological subgroups stratified by stage (A), grade (B), and surgery (C) conditions

Given that the benefit of being single over being DSW varied across some age, race, and HRs subgroups, we conducted analysis to explore whether there were differences of the stage, grade or surgery condition of single patients among these subgroups. As shown in Table 6 and Figure 3, the proportion of stage 0/I, grade 1 and BCS were obviously lower in the <35 years subgroup, in which the protective effect of being single did not occur. What's more, the white race or patients at the ER+/PR + status showed better stage and grade situation, with more BCS accepted than their counterparts.

**Table 6:** Stage, grade, and surgery conditions of patients single at diagnosis in different clinicopathological subgroups (%)

Variable	Stage					Grade					Surgery		
	Stage 0/I	Stage II	Stage III	Stage IV		Grade 1	Grade 2	Grade 3	Grade 4		BCS	Other	No
Age (years)					Age (years)					Age (years)			
<35	23.25	47.59	22.07	7.1	<35	5.53	31.09	61.98	1.41	<35	35.44	57.04	7.53
35‐70	38.99	38.59	16.26	6.16	35‐70	17.08	40.77	41.06	1.09	35‐70	51.94	41.4	6.66
≥70	47.04	34.17	12.38	6.42	≥70	22.7	47.19	29.2	0.91	≥70	52.65	38.87	8.47
Race					Race					Race			
White	42.08	37.37	14.88	5.67	White	19.31	43.38	36.35	0.96	White	52.73	41.16	6.11
Black	29.92	41.43	20.29	8.36	Black	10.65	32.97	54.95	1.43	Black	46.74	43.26	10
AN/AI	32.9	40.39	20.2	6.51	AN/AI	16.94	40.07	42.35	0.65	AN/AI	50.81	45.6	3.58
API	40.32	40.38	14.11	5.18	API	16.7	44.03	38.02	1.25	API	48.48	45.6	5.92
HRs					HRs					HRs			
ER+/PR+	44.27	37.03	13.56	5.15	ER+/PR+	23.05	49.89	26.48	0.58	ER+/PR+	54.79	39.53	5.68
ER+/PR‐	35.34	37.02	18.87	8.77	ER+/PR‐	13.56	39.42	46.07	0.96	ER+/PR‐	46.85	44.43	8.73
ER‐/PR+	26.74	46.14	20.15	6.97	ER‐/PR+	3.77	19.96	73.82	2.45	ER‐/PR+	47.65	41.62	10.73
ER‐/PR‐	26.19	44.29	22.21	7.31	ER‐/PR‐	1.62	16.12	80	2.26	ER‐/PR‐	43.85	47.54	8.61
Other	39.37	35.25	15.31	10.07	Other	17.96	40.44	38.91	2.7	Other	43.49	44.81	11.7

Abbreviations: AN/AI, American Indian/Alaska Native; API, Asian or Pacific Islander; BCS, breast‐conserving surgery; ER, estrogen receptor; HRs, hormone receptor status; PR, progesterone receptor.

## 4 DISCUSSION

In this study, we confirmed what previous studies had shown that marital status impacted on OS of breast cancer patients and we further studied long term cancer‐specific survival of patients. Based on analysis of a large cohort including 298 434 patients and enrolling an integrated range of factors into the multivariate Cox analysis, we found that the benefit of BCSS for being married or single compared with being DSW was consistent with OS, and the association between marital status and BCSS varied by age, race, and HRs. To our knowledge, this is the first study to determine that the effect of marital status on prognosis varies by these demographic and pathological factors, which might reveal the potential mechanism generating this relationship. In further exploration, we found that the variation among these subgroups might be associated with the stage, the grade, and the surgery situation of patients.

A significant difference of OS among three marital subgroups could be observed from the results of 10‐year survival rate and Kaplan‐Meier curves, while the remarkable benefit of BCSS only occurred between the married and other two subgroups. After eliminating the possible interaction among variables with multivariate analysis, we found that single patients showed better BCSS than their DSW counterparts. This result was contrary to our expectation, since single patients were considered lacking for possible social and emotional support from marriage, as well as lower proportion of being at early stage or grade (Table 2). We then explored the effect of marital status on prognosis by different demographic and pathological subgroups, such as age, sex, race, and HRs. Difference of effect of marital status was observed among variable subgroups, and the survival benefit of being single occurred only in several subgroups. Meanwhile, the BCSS benefit of being married over being DSW vanished in AN/AI and male patients. These results hinted that the relationship between marital status and prognosis of patients with breast cancer might be associated with some demographic and pathological factors including age, sex, race, and HRs. Given that there were many more female participants than male, we paid our attention to other three factors. In further analysis of the proportion of stage, grade, and surgery situation in different demographic and pathological subgroups, we found that the BCSS benefit mostly occurred in subgroups showing more stage 0/I, lower grade, and better operation situation.

The first possible underlying reason why married patients with breast cancer had better prognosis is that married patients generally have greater financial resources, which might support them to undergo earlier physical examination, obtain better insurance coverage, and receive more adjuvant therapy.^23^, ^24^, ^25^ Compared with unfavorable marital status and possible financial distress of DSW patients, single patients might also benefit from better economic conditions. Second, undertreatment might also contribute to worse prognosis of the DSW. In this study, we observed that married patients with breast cancer received more surgeries than their counterparts, which indicated that worse prognosis in DSW patients can partly be attributed to undertreatment. Third, psychosocial support may contribute to a better prognosis among married patients with breast cancer. It has been well reported that decreased psychosocial support and greater psychological stress were associated with tumor progression and immune dysfunction. It has also been documented that married patients experienced less depression after diagnosis. This might partly be attributable to the fact that married patients can share the burden of negative emotions and receive psychological support from their spouses.^26^, ^27^, ^28^, ^29^, ^30^, ^31^, ^32^, ^33^ In previous studies of breast cancer, it has been confirmed that social support acted as a predictor of natural killer cell activation.^34^ Moreover, sex hormone disorder induced by psychological factors is closely related to tumor occurrence and development,^27^ which might partly explain why single patients showed better survival than DSW patients, as well as why the BCSS benefit of the single varied across different HRs.

We have suspected that single patients with no experience of marriage and childbearing history might suffer worse survival outcome compared with DSW patients, as what was reported that single patients showed an increased risk of breast cancer.^35^ However, our results of detailed difference among subgroups of marital status indicated that the protective effect of marriage was time‐bound and might be strongly affected by psychological factors. Compared with other counterparts, the worse prognosis of DSW patients might be resulted from negative emotions and worse economic situation due to their unfavorable marital status. In addition, the variation of relationship between marital status and prognosis of patients with breast cancer across race, grade, and HRs indicated that there might be a complicated and comprehensive mechanism underlying, such as genetic factors, hormone levels, function of immune system, psychological factors, social support, and treatment situation. The specific mechanism and its potential clinical practice remain to further research.

Subject to restrictions on our own knowledge and research methods, this study has several limitations. The first is the inherent biases present in any retrospective study. Second, some information relating to both marital status and prognosis of patients with breast cancer was unavailable in the SEER database, such as reproductive history, levels of hormone, and subsequent therapy. Therefore, we were unable to clarify the detailed mechanism of the relationship between marital status and prognosis of patients. Third, marital status was only recorded at diagnosis; details about the duration or quality of the marriage, or any changes in marital status, were not tracked, which might affect the BCSS of patients. Fourth, data of ER and PR status in the SEER database were collected from different local pathology laboratories, which might increase the possibility of bias. Finally, the results of our study are restricted to the United States, as some baseline characteristics such as race, marital status, and surgery history might be different in other countries.

## 5 CONCLUSIONS

Our study demonstrated that married and single patients with breast cancer had better prognosis than their DSW counterparts, which affected by the age, race and HRs. Meanwhile, married patients obtained better survival outcome than single patients. There might be a complicated and comprehensive mechanism underlying the relationship between marital status and prognosis of patients with breast cancer. Further studies should be conducted to clarify the specific influence and mechanism of these factors. According to our research, greater social and psychological support should be provided to DSW patients.

## CONFLICT OF INTEREST

The authors report no conflicts of interest.

## AUTHOR CONTRIBUTIONS

The authors' responsibilities were as follows: Zhen Zhai and Yi Zheng designed the study; Zhijun Dai managed the study; Zhen Zhai, Linghui Zhou and Peng Xu extracted the data; Tian Tian, Pengtao Yang and Shuai Lin performed the analyses; Yujiao Deng, Cong Dai, Qian Hao and Na Li interpreted the evidence and wrote the manuscript; Hongtao Li and Zhijun Dai revised the article. All authors agreed to be accountable for the work.
